# Advancing nutrition science to meet evolving global health needs

**DOI:** 10.1007/s00394-023-03276-9

**Published:** 2023-11-28

**Authors:** Lynnette M. Neufeld, Emily Ho, Rima Obeid, Charalampos Tzoulis, Marina Green, Luke G. Huber, Michelle Stout, James C. Griffiths

**Affiliations:** 1 Food and Nutrition Division, Food and Agriculture Organization of the United Nations, Rome, Italy; 2 Linus Pauling Institute and College of Health, Oregon State University, Corvallis, OR USA; 3 Department of Clinical Chemistry and Laboratory Medicine, University Hospital of the Saarland, Homburg, Germany; 4 Department of Neurology, Haukeland University Hospital, Bergen, Norway; 5 Department of Clinical Medicine, K.G. Jebsen Center for Translational Research in Parkinson’s Disease, University of Bergen, Bergen, Norway; 6 Nutrition Research Centre Ireland, South East Technological University, Waterford, Ireland; 7 Council for Responsible Nutrition, Washington, DC USA; 8 Amway/Nutrilite, Buena Park, CA USA; 9 Council for Responsible Nutrition–International, Washington, DC USA

**Keywords:** Choline, NAD-replenishment, Nutrition, Omega-3 fatty acids, Public health, Xanthophyll carotenoids

## Abstract

Populations in crisis!

A global overview of health challenges and policy efforts within the scope of current nutrition issues, from persistent forms of undernutrition, including micronutrient deficiency, to diet-related chronic diseases. Nutrition science has evolved from a therapeutic and prevention emphasis to include a focus on diets and food systems. Working and consensus definitions are needed, as well as guidance related to healthy diets and the emerging issues that require further research and consensus building. Between nutrient deficiency and chronic disease, nutrition has evolved from focusing exclusively on the extremes of overt nutrient deficiency and chronic disease prevention, to equipping bodies with the ability to cope with physiologic, metabolic, and psychological stress. Just what is ‘optimal nutrition’, is that a valid public health goal, and what terminology is being provided by the nutrition science community? Nutrition research on ‘healthspan’, resilience, and intrinsic capacity may provide evidence to support optimal nutrition. Finally, experts provide views on ongoing challenges of achieving consensus or acceptance of the various definitions and interventions for health promotion, and how these can inform government health policies.

Nutrition topics that receive particular focus in these proceedings include choline, NAD-replenishment in neurodegenerative diseases, and xanthophyll carotenoids. Choline is a crucial nutrient essential for cellular metabolism, requiring consumption from foods or supplements due to inadequate endogenous synthesis. Maternal choline intake is vital for fetal and infant development to prevent neural tube defects. Neurodegenerative diseases pose a growing health challenge, lacking effective therapies. Nutrition, including NAD-replenishing nutrients, might aid prevention. Emerging research indicates xanthophyll carotenoids enhance vision and cognition, potentially impacting age-related diseases.

## Introduction

Poor and suboptimal nutrition is a global issue that affects a significant portion of the population, particularly preschool children, and women of reproductive age. Despite some progress being made in reducing poor nutrition, the COVID-19 pandemic and rising food prices may have impeded this progress. Micronutrient deficiency, while not generally included in global targets, is a major concern. Healthy diets are essential for preventing disease and promoting optimal health, but there is often contradictory advice, especially in the lay press and on social media platforms, on what constitutes a healthy diet. This report explores the role of nutrition in optimizing human health, including the importance of choline, NAD (nicotinamide adenine dinucleotide) replenishment in neurodegenerative diseases, and the xanthophyll carotenoids.

Precision and personalized nutrition, which consider individual differences in response to food, nutrients, and bioactives, is an emerging area of opportunity. The microbiome, which plays a key role in human health and how we metabolize nutrients and respond to food, has implications for personalized nutrition, as diets tailored to individual microbiomes may optimize health.

Choline is an essential nutrient that plays a vital role in cell metabolism and functions. It is not synthesized by the body in adequate amounts, making it necessary to consume choline-rich foods or supplements. The importance of maternal choline supply in fetal and infant development has been highlighted in the literature, with low dietary choline intake or low circulating levels in the mother being associated with an increased risk for neural tube defects. Choline deficient diets can cause fats to accumulate in the liver, and removing choline from the diet causes fatty liver in preclinical research. As such, public health authorities across the globe should recognize choline as an essential nutrient for early life development.

Neurodegenerative diseases (ND) are a major health challenge in the twenty-first century, with the number of people affected expected to continue to substantially increase in the coming decades. Currently, there is a lack of neuroprotective or disease-modifying therapies available to prevent or delay disease progression. Primary prevention would be a much more efficient approach than treatment, and population-wide prevention would be an ideal approach against ND. Diet, including nutrients for NAD replenishment, may play a role in the prevention of ND.

The xanthophyll carotenoids (XC), lutein (L), zeaxanthin (Z), and meso-zeaxanthin (MZ) are natural lipid-soluble micronutrients obtained only from the diet. They have become increasingly important for their role in preserving and enhancing human function, such as visual performance and potentially cognitive function, along with their potential diagnostic and therapeutic implications for chronic and age-related diseases. Understanding the underlying mechanisms by which they are absorbed and metabolized is important for developing targeted nutrition as a cornerstone for individualized medicine.

Poor nutrition may appear as undernutrition, micronutrient deficiencies, and as diet-related non-communicable diseases. Each of these situations has the potential to lead to severe disease states as well as social and economic burdens. It is a public health imperative to implement policies that address these modifiable challenges and support access to healthy diets worldwide. In addition, developments in precision and personalized nutrition advance the understanding of responses to food, nutrients, and bioactives, leading to improved health outcomes. This CRN-International Scientific Symposium and resulting conference report are intended to support the path toward a nutrition policy roadmap that will improve the health of current and future generations.

Over the last decade, the Council for Responsible Nutrition-International (CRN-I) has focused increasingly on several over-arching issues at the annual CRN-I Scientific Symposium and concomitant publications in the European Journal of Nutrition. The symposia have been held at the Codex Alimentarius Committee on Nutrition and Foods for Special Dietary Uses (CCNFSDU). The most recent topics are tangential to each other and have covered optimal nutrition [46, 78, 131], healthy ageing [79, 101], and concepts around health promotion[57]. Further key publications that explore these inter-related topics include: *From Lifespan to Healthspan *[153]; *Opportunities to Improve Nutritional Status and Promote Health *[102]; *Sex Differences Across the Life Course*[6] and *Optimizing Health with Nutrition-Opportunities*[63].

## From nutrient deficiencies to chronic disease: Evolving evidence of nutrition and the role of healthy diet in crisis

Malnutrition remains an important public health problem in most parts of the world, with important variability in the forms of malnutrition and progress to address it.

The world is off-track to meet most globally agreed nutrition goals, such as the World Health Assembly Targets, and the nutrition-related Sustainable Development Goals (SDGs) [45]. At the global level, progress was being made for several forms of undernutrition but evidence suggests that the COVID-19 pandemic and recent increasing food price crises may be reversing this trend [45]. Unfortunately, global targets do not include all critical forms of malnutrition, for example micronutrient deficiency. Recent global and regional estimates suggest that globally, 56% of pre-school aged children, and 69% of women of reproductive age are deficient in one or more essential micronutrients [139]. That study also highlighted that no global region is free from micronutrient deficiency in these population groups. Many countries face issues of overweight and obesity simultaneously with several forms of undernutrition – at national (both ends of the spectrum in separate populations in the country), household (e.g., obese adult and undernourished child), and/ or individual (e.g., obese individuals with micronutrient malnutrition) levels [117, 120].

Global monitoring and national survey data mask important inequities in the rate and extent of progress to address malnutrition [149]. Such monitoring data also mask important inequities with rural residence, lower education, lower economic status, and minority ethnic status as strong predictors of several forms of malnutrition in many countries globally, for example illustrated by data from Latin America [8]. Raising awareness of these issues and ensuring appropriately targeted approaches to address all forms of malnutrition is constrained by critical data gaps, for example for micronutrient status [25]. There are also important issues to resolve in the quality of the measures used to assess nutritional status at the population level. One notable example is the unresolved issue of large differences in hemoglobin concentration measured by venous and capillary blood samples. These differences suggest that surveys using capillary blood may be substantially overestimating the burden of anemia [110, 122].

Human survival, health, growth, development, and well-being – like all systems – have many drivers and determinants [146]. It has been well demonstrated that the consequences of undernutrition are intergenerational, i.e., that the nutritional status of the mother in her own infancy and childhood influences fetal growth and development and related nutrition of subsequent generations as well as other health-related issues. These issues in turn affect adult health, physical and cognitive functioning and ultimately the development of society [132]. Diet-related, non-communicable diseases similarly limit human capacity and place undue burden on health care systems [72].

Unhealthy diets are a common cause of all forms of malnutrition [144] and a major risk factor for death and many adverse health outcomes, including non-communicable diseases [53]. There are however, many data issues that limit the ability to generate robust estimates of these diet-disease relationships [9]. For example, the Global Burden of Disease (GBD) estimates include individual-level dietary intake data when available but extrapolate from national and household data to fill gaps. Given the known issues of inequity, there are considerable limitations to assume national and household data can reflect individual intake [9]. Similarly the disease estimates from these analyses include a variety of studies including randomized controlled trials, but also observational studies and short-term studies measuring immediate rather than long-term disease endpoints [9].

**Fig. 1:**
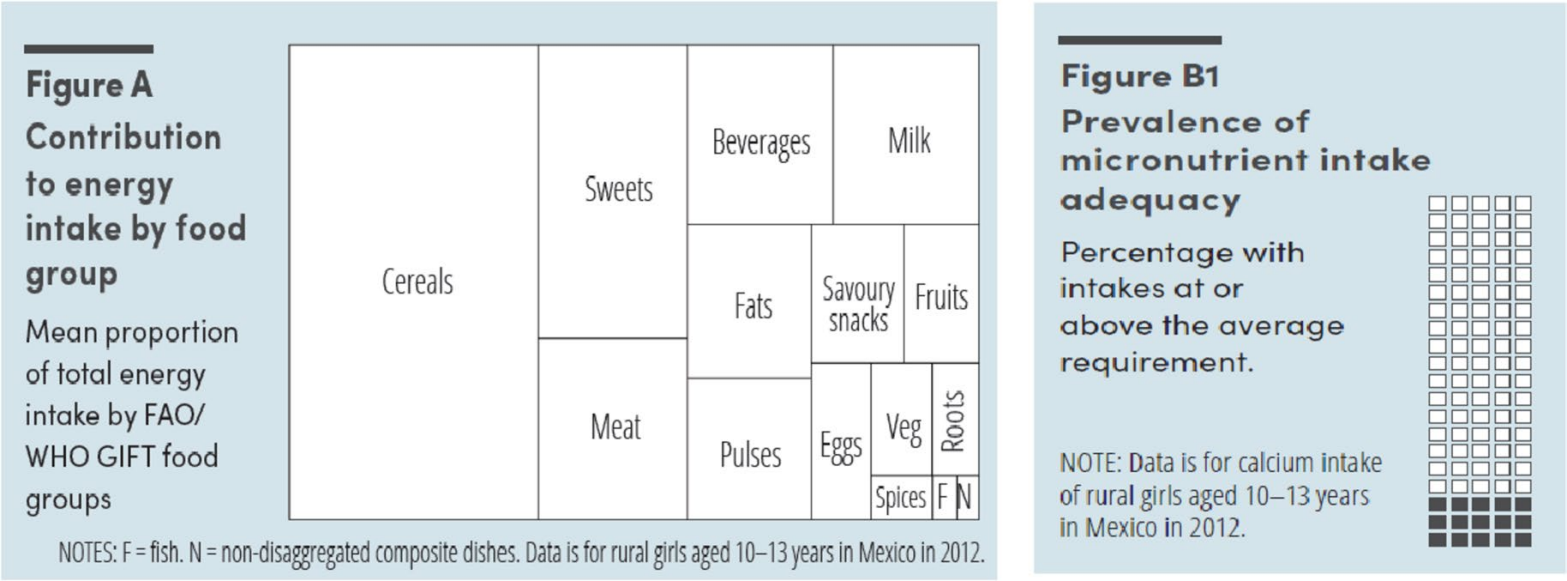
Possible data visualizations from quantitative individual level dietary data [44]

At least in part, the inability to generate more robust estimates of the disease burden of unhealthy diets relates to data gaps. Quantitative dietary intake data permit in-depth understanding of dietary patterns and permit the assessment of all components of what constitutes a healthy diet, including nutrient adequacy, consumption of health-promoting foods, and moderation in consumption of unhealthy foods. Such data enable robust study of diet and health associations and are vital to inform policies and programs (Fig. 1). Unfortunately, few countries have recent quantitative individual-level dietary intake data [44] and information on the determinants of dietary intake. This is particularly problematic during critical life stages such as adolescence [109].

The poor availability of dietary intake data may be due to many factors, including the current high level of effort and resources required for its collection and analysis. There have been a number of advances on the development and validation of metrics to assess some dietary components [24, 160]. Highly simplified methods that prioritize cross-context comparability have been developed that may be appropriate for global monitoring [62], but many gaps remain in the validation of such instruments across contexts [148].

At the same time, there is an abundance of advice, often contradictory in the scientific literature and public media on what constitutes a healthy diet. Resolving apparent contradictions and providing appropriate guidance (to government, the food industry, the public) requires consensus on what constitutes a healthy diet and how to measure it. These issues have been identified and efforts are underway to address them [43]. Publications that will provide robust review of the evidence on what constitutes a healthy diet and provide a roadmap to reach consensus on how to measure it are forthcoming.

## Defining optimal health

**Fig. 2:**
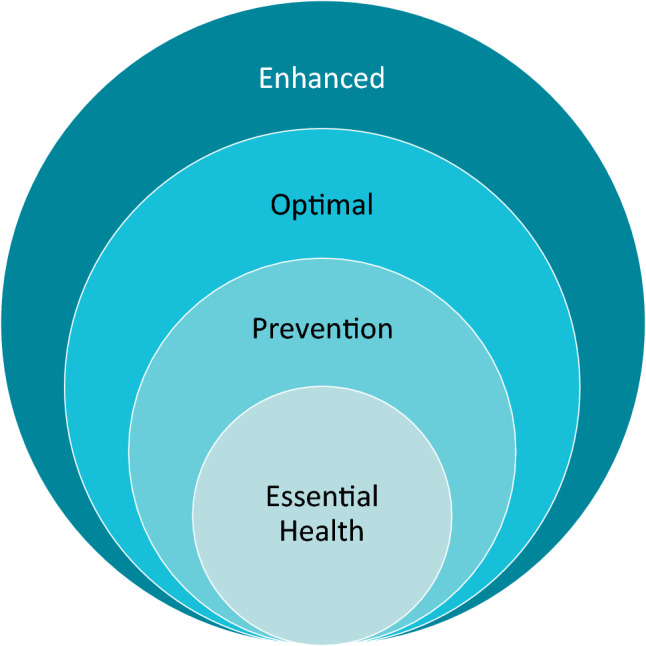
Emerging needs for framing dietary requirements from the prevention of overt nutrient deficiencies to optimal health

Over the last many decades, there is increasing evidence of the impact that nutrition has on human health. Deficiencies in essential micronutrients have widespread and varied impact on health – including impairing growth and development, compromising immune function, and affecting an individual’s ability to thrive and meet their potential. Although we continue to define the essential roles that nutrients play as energy sources, cellular structure components, enzyme cofactors and signaling molecules, there is a strong need to re-frame and adapt nutrition recommendations from simply preventing nutrient deficiencies to supporting optimal health (Fig. 2). Indeed, as the population is living longer, an emerging focus for nutrition will be maintaining optimal health over the life course.

Now more than ever in our history, our population’s health has emerged as a global priority. Currently, 6 in 10 adults in the US have a chronic disease, and 4 in 10 have two or more [108]. The number of older adults is projected to double from 52 million in 2018 to 95 million by 2060 [145]. By 2030, 1 in 5 Americans is projected to be 65 years old or over. As the major risk factor for many chronic illnesses is age, it is anticipated that the rates of all age-related diseases, especially chronic diseases, will skyrocket, potentially overwhelming the healthcare system. We need to enable the healthcare system and the population to be more proactive rather than reactive towards health outcomes. There is a critical need to help find solutions to optimize our healthspan; where we support and enable living better longer. The “force of biological aging” (senescence) drives diseases and in turn, our lifespan. However, disease incidence rises quicker than our average lifespan, which suggests that the quality of life begins to decline faster than our lifespans. Ensuring optimal nutrition is a significant and easily modifiable variable in the solution for maintaining and improving healthspan. To fully realize the vision of optimal health, there exist several challenges and gaps, but there are also recent advances and exciting opportunities.

Malnutrition and meeting nutritional needs are a global issue, populations are food insecure, but even in food secure regions – malnutrition still exists as “hidden” hunger, where essential micronutrient needs are not being met. The global prevalence of deficiency in at least one of three micronutrients is estimated to be 56% among preschool-aged children and 69% among non-pregnant women of reproductive age [139]. In North America, the Dietary Reference Intakes (DRIs) for individual nutrients, including the Estimated Average Requirement (EAR) and the Recommended Dietary Allowance (RDA), are life stage- and sex-specific recommendations for Americans and Canadians. These reference intakes were established in the 1990s by the Food and Nutrition Board of the National Academies of Sciences, Engineering, and Medicine to prevent deficiency disease and to reduce the risk of chronic diseases [70]. However, incorporating chronic disease endpoints has been extremely challenging, primarily because data are largely lacking. Such endpoints were used to set the DRIs for only a handful of nutrients [104]. Thus, the current DRIs, including the RDAs that are aimed to cover the nutrient needs of 98% of the population, do not account for the amount of a nutrient that one needs in order to achieve and maintain ‘optimal’ health.

One key barrier to defining optimal health include a lack of sensitive and specific biomarkers for nutrients. There is a critical need for better biomarkers for several nutrients – especially those that are homeostatically regulated. We often rely on self-report dietary recalls or food frequency surveys to assess nutrient status; these tools have inherent flaws and biases that limit their accuracy and precision. For example, we lack a sensitive and specific biomarker for zinc intake [83, 84], which is one of the top global micronutrient deficiencies [82]. Data from several laboratories have established that fasting plasma zinc levels (the current clinical test) is not highly responsive to changes in zinc intake [137, 140]. Importantly, functional consequences of low zinc intake, such as increased oxidative stress, increased DNA damage and compromised immune function can be evident even when there is no change in plasma levels [64, 77]. This highlights the need to identify better biomarkers of zinc intake that precede these functional health consequences. In addition to the needs for better biomarkers of essential micronutrients, there are numerous non-essential bioactives and natural compounds that will contribute to optimal health that will also need status measures.

A second gap is the need to identify better biomarkers of healthspan and optimal health. There has been considerable interest in identifying markers of biological versus chronological age. Horvath has proposed an epigenetic clock as a framework to assess biological age [66]. Several other epigenetic clocks have also been proposed [92, 96]. There is also interest in other functional biomarkers of healthspan and biological age. The mitochondria are vital cell organelles that coordinate the biochemical conversion of latent dietary “fuel” macromolecules into a usable metabolic energy. Many vitamins and minerals exert their effects as coenzymes and cofactors for mitochondrial energy transduction or help maintain mitochondrial function by preventing oxidative damage. Thus, deficits in any vitamin or mineral has a profound effect on mitochondrial function and overall healthy aging [150]. Researchers have proposed using mitochondrial function and a “Cell Bioenergetics Health Index” as an alternative functional index of healthy aging [28]. We also know that immune dysfunction is another functional consequence of several micronutrient deficiencies and age. In particular, chronic inflammation is a consequence of age and precursor to several chronic disease states. More recently researchers at the Buck Institute have developed an Inflammatory Age (iAge) Clock that utilizes deep immune profiling and various clinical assessments to assess biological age [127]. More research is needed to validate these emerging tools to assess biological age and healthspan.

A third gap, and opportunity, centers around the concept of ‘precision nutrition’ in defining optimal health. To realize the promise of optimal health, we need to appreciate that one size does not fit all. Understanding the drivers of differential and individualized responses to food, nutrients, and bioactives will be critical to realize the potential of precision nutrition. There is a critical need to understand the interactions among age, gender, environment, and genetics on how an individual responds to factors derived from foods. Emerging areas, including the roles of genotype, metabolism, and the microbiome are significant areas of opportunity. The role of genotype and single nucleotide polymorphisms has been long established. Some classic examples include polymorphisms in folate and single carbon metabolism that alters individual micronutrient metabolism and increases the susceptibility to disease [55, 151]. Individual genetic variation can influence how nutrients are assimilated, metabolized, stored, and excreted. However, a growing body of evidence implicates the microbiome as playing a key role in human health [81, 86, 119]. Increasing evidence that the microbiome may also be determinant of individual variation is also clear. The microbiome may be a key determinant and personalizing factor that determines how well you metabolize nutrients in food, and ultimately affect how well they work for you and your health. The Ho laboratory found that an individual’s microbiome may be a determining factor in an individual’s capacity to metabolize cruciferous vegetables-derived bioactives, such as sulforaphane [20, 21]. In these studies, there is wide variation in the production of the biologically-inert microbial-derived metabolite, sulforaphane-nitrile and highlights the possibility that the microbiome could determine the metabolism and efficacy of health responses to cruciferous vegetable intake. As the field moves toward realizing the vision of personalized nutrition with genotype, metabotypes, and the microbiome in mind, it will aid in enabling nutrition guidelines that can be more individualized, and also addressing both preventing deficiencies and optimizing health (Fig. 2).

## Choline during pregnancy and lactation and fetal and infant development

Choline is an essential nutrient for adults because it is not synthesized by the body in an amount that is adequate to cover the daily needs [71, 162]. A diet deprived of choline causes liver steatosis, while adding choline to the diet can revert this phenotype [162]. Choline has three distinct roles in cell metabolism and functions. First, choline is a methyl donor in one carbon metabolism after it is oxidized to betaine. Thus, choline interacts with folate and vitamin B12 and can lower plasma homocysteine. Second, phosphatidylcholine is synthesized in the liver and muscles from choline or phosphatidylethanolamine. Phosphatidylcholine is an essential component of all cell membranes and blood lipoprotein particles that circulate lipids in a soluble form between blood and tissues. Third, choline is a source of acetylcholine, a neurotransmitter with key functions in neuronal cells.

The role of the maternal choline supply in fetal and infant development has gained more attention in recent years [114, 161]. The effect of adequate maternal folate intake (e.g., folic acid) during early pregnancy on reducing the risk of neural tube defects (NTDs) is established [31, 33, 115] and it has been confirmed by randomized controlled trials and observational data from countries applying mandatory folic acid fortification [107]. An average risk reduction of 50% has been reported, implying that 50% of the risk is not explained by folate insufficiency and thus, not preventable by increasing folate intake. This raises the question on whether insufficient intake of choline may increase the risk of NTDs and whether higher choline intake or choline supplementation may be an additional nutrient to further reduce the risk of NTDs.

A recent systematic review and meta-analysis of human studies published from 1997 to 2021 investigated the association between maternal choline intake and the risk of NTD or outcomes related to neurodevelopment of the infant or child [114]. Low dietary choline intake, or low circulating levels in the mother, was associated with a higher odds ratio for NTD [pooled estimate (95% confidence intervals) = 1.36 (1.11, 1.67); 95% prediction intervals = 0.78, 2.36][114]. Higher maternal choline intakes during the second half of pregnancy and early postnatal period (550 mg up to 1 g/d on top of the diet), or a child intake of 513–625 mg/d from supplements were safe and likely to demonstrate favorable effects on several domains of child neurocognition such as memory, attention and visuospatial learning versus the comparators [114].

Choline-deficient diet causes fats to accumulate in the liver [27]. This universal effect has been consistently reported in experimental depletion-repletion studies on different species including humans [12, 65, 118, 159]. The mode of action in humans and other animals is similar. Dietary choline is a source of phospholipids that contribute to removing triglycerides from the liver. Animal studies have unambiguously shown that isotope-labelled choline added to the diet of the mother can be detected in the liver of the fetus [50]. Human studies have shown that concentrations of choline are high in cord blood and breastmilk confirming that the fetus and infants obtain their choline from the mother [69, 106]. Pregnancy and lactation are associated with triglyceride accumulation in the liver of the mother [37, 163]. Removing choline from the diet of pregnant rats causes fatty liver not only in the mother, but also in the fetus [103]. A choline-deficient diet fed to newborn pigs also caused fatty liver [74]. Therefore, the fetus and newborn cannot synthesize choline in amounts that are necessary to maintain normal liver function. Recently, the European Food Safety Authority (EFSA) approved a health claim on choline and contribution to normal liver function of the fetus and exclusively breastfed infants (according to Article 14 of Regulation (EC) No 1924/2006) [41]. According to the EFSA Panel, the scientific evidence has shown that ‘Maternal choline intake during pregnancy and lactation contributes to normal liver function of the fetus and exclusively breastfed infants. Future studies in humans need to address the effect of maternal choline intake during pregnancy on additional endpoints such as gestational diabetes, fatty liver in the mother, and the risk of congenital heart defects.

From a public health perspective, meeting the present recommendations for choline intake during pregnancy and lactation [40, 71] is important for normal liver function and could offer an opportunity to further reduce the risk of NTDs and mitigate risk factors of impaired child neurocognitive development. The window of opportunity to modify these outcomes is rather short. The critical periods are during the whole pregnancy and lactation (i.e., infant age 6–12 months). Therefore, authorities are just starting to recognize the role of choline in fetal and infant development. There is a need to translate the science around choline into strong recommendations and increase awareness of gynecologists and women of pregnancy age for this nutrient. Moreover, prenatal multivitamin supplements should contain choline in addition to folate and other key nutrients.

## NAD-replenishment and dietary modification as neuroprotective strategies against neurodegeneration

Neuodegenerative diseases (ND) constitute one of the biggest and most rapidly growing challenges facing healthcare and society. As the population ages, the incidence of Alzheimer disease (AD), Parkinson’s disease (PD), dementia with Lewy bodies (DLB), and related disorders rises dramatically, with the number of people affected by ND expected to exceed 150 million by 2050 [51, 52, 54]. The combined cost in Europe for the two most common neurodegenerative disorders, dementia, and PD, was estimated at €119 billion in 2010, which is at least equal to that of cancer [15, 52]. There are currently no neuroprotective or disease-modifying therapies able to prevent ND, or to delay disease progression. As a result, affected individuals face a future of progressive motor and cognitive disability, early institutionalization, and premature mortality [15, 51, 52, 54, 85].

An important limitation to developing successful neuroprotective therapies for ND is the fact that we are not able to initiate treatment interventions early enough in the course of the disease. The process of neuronal dysfunction and degeneration starts years or even decades before the onset of symptoms that herald the diagnosis [38, 113, 157]. This means that at the time of diagnosis, individuals have already suffered severe and irreversible neurodegeneration. Thus, primary prevention would be a much more efficient approach than treatment, in ND. Moreover, since we currently lack biomarkers that are sufficiently sensitive, specific, and broadly applicable, to allow us to confidently detect individuals early enough, at preclinical stages of ND, population-wide prevention would be an ideal approach. In addition, effective prevention of ND would be much more cost-effective than treatment. Treating neurodegenerative diseases is extremely costly, both for individuals, healthcare systems, and society as a whole [35, 116]. By investing in preventive measures, the overall healthcare costs can be significantly reduced.

Appropriate primary prevention for ND should be safe, tolerable, easy to administer, and relatively low-cost, so that it may be implemented globally, on a population-wide level. Thus, dietary modification, including the use of appropriate nutritional supplementation, would be an ideal measure towards this goal. The pertinent question is *what kind of dietary supplementation could help prevent or delay ND?* Several micronutrient compounds have shown promise in recent years, including carotenoids (reviewed elsewhere in this article) and compounds increasing the levels of nicotinamide adenine dinucleotide (NAD).

NAD, which constantly shuttles between its oxidized (NAD^+^) and reduced (NADH) state, is a vital cofactor for metabolic redox reactions, including mitochondrial respiration. In addition, NAD^+^ is required as substrate for a multitude of essential signaling reactions involved in DNA repair, protein deacylation, inflammation, and second messenger generation [80]. Cellular NAD levels decline with age [76, 80], and increasing the NAD-replenishment rate via supplementation of precursors has shown beneficial effects on life- and healthspan in multiple animal models, and strong evidence of neuroprotection against ND [22, 76, 80, 129]. A unique and intriguing feature of NAD-replenishment is that it targets multiple processes associated with ND, including impaired mitochondrial metabolism and bioenergetics, accumulation of somatic DNA damage, dysregulated epigenomics, declining lysosomal and proteasomal function, and neuroinflammation [23, 89, 142, 147]. In this manner, NAD-replenishment may increase neuronal resilience, shielding neurons against multiple forms of disease-associated stress [89, 147]. Therefore, NAD-replenishment may be a viable therapeutic approach across ND.

NAD can be replenished via supplementation of precursors, such as the vitamin B3 forms; nicotinic acid, nicotinamide, and nicotinamide riboside (NR) [13, 80]. These compounds have been extensively tested in preclinical and clinical studies and have been shown to be generally well tolerated by adult humans, with no evidence of medically unacceptable toxicity with currently tested dose regimens [23, 36, 48, 147]. The *NADPARK* study [23, 61], a recent clinical trial of NR on newly diagnosed Parkinson’s disease, produced highly encouraging results, showing that oral NR at a dose of 1000 mg daily significantly increased NAD levels in the human brain, and this was associated with altered cerebral metabolism and a mild but significant clinical improvement. Moreover, NR was associated with a number of beneficial metabolic effects, including a systemic augmentation of the NAD-metabolome, and upregulation of pathways integral to mitochondrial respiration and proteostasis, both hallmark pathogenic processes in parkinsonism [60].

Diet plays a role in ND [130, 158], and dietary and nutritional modification may contribute to a preventive strategy against these diseases. However, to be able to contribute in addressing the challenge of neurodegeneration, nutrition policy should evolve in several key ways:

1. Emphasize the importance of a healthy, balanced diet. While not much is known about how dietary factors influence the risk of ND, increasing evidence suggests that a healthy and balanced diet that is rich in fruits, vegetables, whole grains, lean proteins, healthy fats, and micronutrients, may contribute to modifying the risk of ND [19, 68, 124, 130, 158]. The Mediterranean diet in particular has been associated with a decreased risk of Parkinson’s and Alzheimer’s disease [1, 5]. Moreover, such a diet will at the very least allow individuals to better cope with ND, by giving them a base of good general health. Thus, nutrition policies should prioritize the promotion of a healthy and balanced diet, such as the Mediterranean diet [1, 5].
2. Address micronutrient deficiencies. Certain micronutrients, including but not limited to, vitamin E [143], vitamin D [73], carotenoids [130], niacin and other precursors of nicotinamide adenine dinucleotide (NAD) [23, 147], play crucial roles in brain health and may have a protective effect against neurodegenerative diseases. Nutrition policies should focus on identifying and addressing micronutrient deficiencies in populations through strategies such as food fortification, public health campaigns, and targeted supplementation programs.
3. Support early life nutrition. Early life nutrition plays a critical role in brain development and may have long-term implications for neurodegeneration risk via a number of molecular mechanisms, including epigenetics [11, 49]. Policies should, therefore, focus on supporting and improving access to nutrient-rich foods for pregnant and lactating women, and young children, and implementing nutrition education programs targeting parents and caregivers.
4. Advocate against the use of pesticides of proven or tentative neurotoxicity. Exposure to numbers of pesticides inducing free radical generation and/or mitochondrial respiratory deficiency, such as rotenone and paraquat, has been associated with an increased risk of Parkinson’s disease [121]. It is therefore important that nutrition policies and legislation make it a priority to increase the knowledge regarding these risks and to prohibit the use of pesticides that are not proven to be safe.
5. Encourage research and education. To harness nutritional and dietary modification in the prevention of ND, we need to greatly expand the knowledge of how nutrition influences the risk of ND. Thus, nutrition policies should prioritize research funding and support initiatives that further our understanding of the relationship between nutrition and ND. This includes supporting basic and translational science, observational cohort studies, and clinical trials, to gather more evidence on the impact of specific nutrients (or the lack of) and dietary patterns on brain health. Additionally, educational campaigns can raise awareness about the importance of nutrition in brain health and provide practical guidance on making healthier food choices.

Overall, evolving nutrition policy to address the challenge of neurodegeneration requires a multifaceted approach that emphasizes healthy dietary patterns, reduces consumption of unhealthy foods, addresses micronutrient deficiencies, promotes specific nutrients beneficial to brain health, supports early life nutrition, avoids the use of chemicals increasing the risk of ND, and encourages research and education.

## The xanthophyll carotenoids: Clinical applications and targeted nutrition

Chronic and degenerative diseases have become a burden for medical science and public health. Inadequate lifestyle choices and nutrition are the key risk factors leading to these conditions. Unfortunately, we are facing an era of deficient nutrition and inappropriate dietary patterns. Robust evidence on micronutrients is essential to gain an in-depth knowledge of their metabolic and functional roles to inform recommendations that guide human wellbeing. The main focus in this section is a group of micronutrients, the xanthophyll carotenoids (XC), lutein (L), zeaxanthin (Z), and meso-zeaxanthin (MZ), their bioavailability, and their importance in diet and nutraceuticals as health-enhancers and disease-modifiers.

The XC are natural lipid soluble micronutrients that belong to the xanthophyll class of the carotenoid family. They are obtained only from diet and have become increasingly important for their role in preserving and enhancing human function, such as visual performance and cognitive function, along with the potential implications for diagnosing and managing chronic and age-related diseases. Understanding the underlying mechanisms by which they are absorbed and metabolized are the bases for developing targeted nutrition as a cornerstone for individualized medicine.

This section addresses L, Z, and MZ effects in the human body from a practical standpoint, in terms of nutrient concentrations, function, metabolism, dietary interactions and lifestyle determinants. Furthermore, it explores the needs and challenges for their application in clinical practice, and how we can implement these research findings for future recommendations in health care and nutrition.

Chronic and degenerative diseases have become an important burden for medical science and public health. Lifestyle and nutrition are the most important risk factors leading to these conditions. Unfortunately, we are facing an era of deficient nutrition [93] and inappropriate dietary patterns [105]. Current recommendations [138] for a healthy diet have been shown to be suboptimal in providing adequate concentrations and varieties of micronutrients [155]. Despite increased longevity and decreased age-specific death rates, chronic and degenerative diseases remain highly prevalent and expensive to treat [7, 32, 88, 128, 133]. In the quest to overcome these challenges, we must acknowledge the relevance of nutrition in health and disease. Robust evidence on micronutrients is essential to gain in-depth knowledge of their metabolic and functional roles to guide government and public health recommendations to shape human wellbeing.

**Fig. 3:**
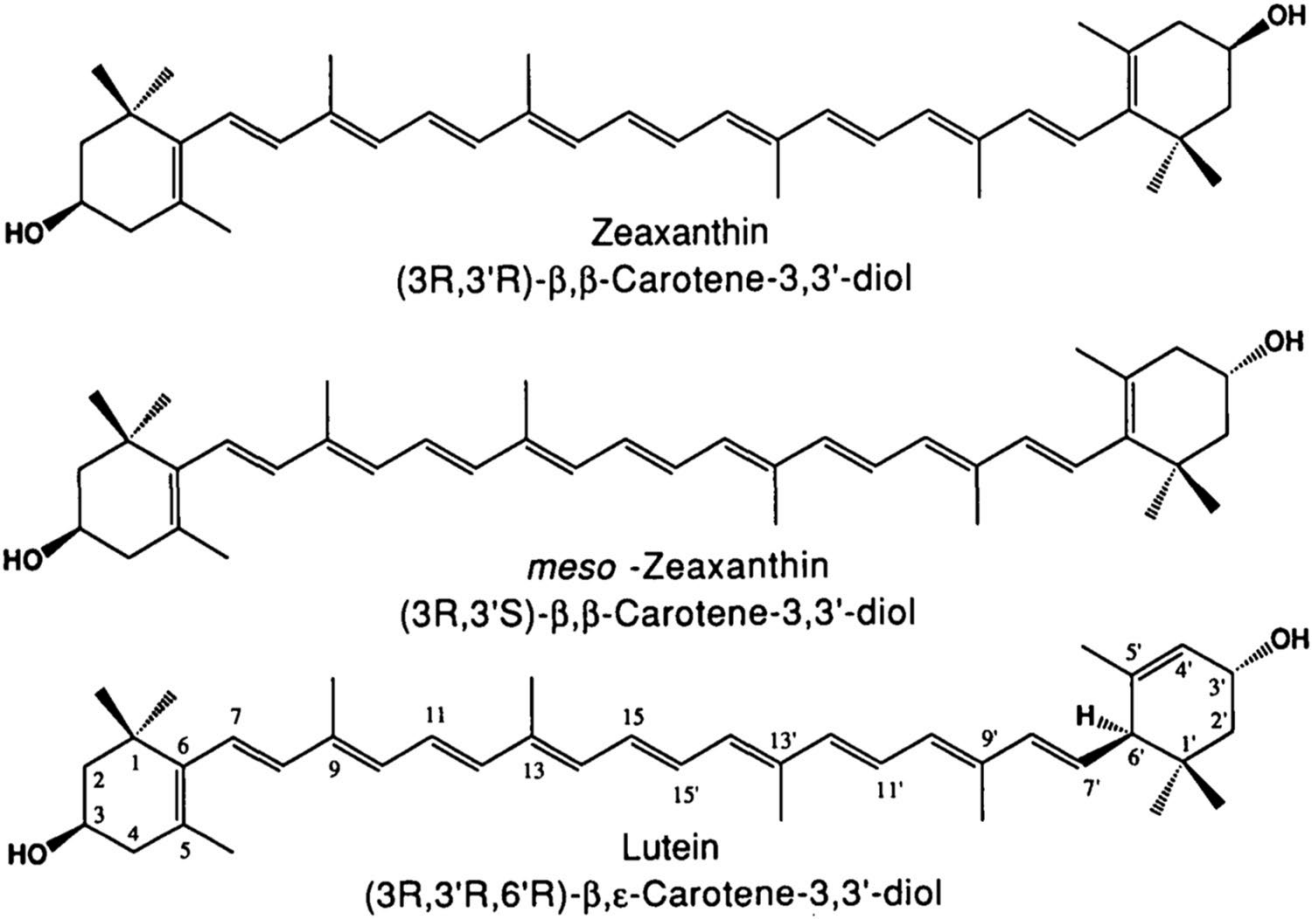
The chemical structure of the xanthophyll carotenoids [18]

Carotenoids, a diverse group of micronutrients found in various fruits and vegetables, have long been recognized for their essential role in human health. Among these carotenoids, the xanthophyll carotenoids (XC), namely lutein (L), zeaxanthin (Z), and meso-zeaxanthin (MZ), natural lipid soluble pigments [156] stand out due to their unique chemical structure (Fig. 3) and potent biological activities. L, Z, and MZ have a high capacity to absorb short-wavelength visible light, making them excellent short-wavelength filters [135], offering protection against light-induced oxidative damage. They are obtained only from the diet [111] and have become increasingly important for their clinical applications and targeted nutritional intervention [30], preserving and enhancing vision [4] and their implications in the medical field [29]. The latest report of the renowned AREDS study, detailed a decreased risk of progression to late age-related macular degeneration (AMD) in a sample of over 4000 patients, supplemented with L and Z (along with other antioxidants) [3]. Additionally, there is mounting evidence linking xanthophyll carotenoids to cardiovascular protection. Howard and Thurnham suggested that the protective effects of vegetables and fruit against cardiovascular disease (CVD) may be in part through L intake, which may reduce tissue oxidation and prevent activation of damaging complement factors [67]. Furthermore, L supplements have been shown to reduce plasma complement factors, including the membrane attack complex of the complement system [141].

Diseases have multifactorial influences, from genetic, epigenetic, metabolic, and environmental. Therefore, it is necessary to consider a multidisciplinary approach to treat a disease, and more importantly, to preserve health. Currently, there is limited understanding of the effects and interactions of micronutrients in terms of regulation, structure, and function in the human system. This, together with the challenge of proving causal relationships in nutrition, is the greatest barrier for its clinical application.

There are two outstanding characteristics of the XC: (1) their highly unsaturated, conjugated chromophore structure responsible for their physicochemical and biological properties [47] and (2) their hydrophobic character, conditioning poor aqueous solubility in the native state and in turn low bioavailability when ingested in the human diet [87]. XC behave as potent antioxidants [42] in biological tissues (e.g., retina) and portray other biological properties such as photo-induced damage protection [134], membrane stability [58, 154], and putative anti-inflammatory effects [91, 100].

L, Z, and MZ have been the focus of research and commercial interest for their benefits to human physiology and metabolism. In addition, we are currently facing a time of deficient nutrition for a myriad of reasons, with devolution theory [34, 94] and inappropriate dietary patterns being key factors. Therefore, L, Z, and MZ derivatives with increased hydrophilic character are valued for their enhanced biological activity [59]. Of note, novel hydrophilic carotenoid-derivatives with L, Z, and MZ diacetates have shown improved bioavailability and metabolism in tissue in supplemented healthy participants [56].

Identifying micronutrient biomarkers by measuring structure or function add value to their role in health and disease, and therefore, in clinical use. Evidently, serum concentrations are the gold standard to quantify human metabolism for any given nutrient. Interestingly, L, Z, and MZ singularly deposit in the macula lutea [123], where they are known as macular pigment (MP). Remarkably, the MP provides a unique opportunity to be measured non-invasively, enabling human nutrition to be quantified at a tissue level. The significance of measuring MP is supported by scientific evidence demonstrating its protective capacity against a spectrum of degenerative diseases. This protective role extends from specific macular pathologies such as AMD [10, 126, 152] to broader age-related diseases like dementia [112].

Regarding L and Z dietary intake, food content is only a contributor to the total intake of these micronutrients. In addition to food content, dietary intake of L + Z in humans depends on factors such as race and ethnicity [75], which determine intake frequency and dietary patters (i.e., meal composition[99]), and impact on the final consumption of L + Z in diet. Furthermore, XC dietary intake is limited by their bioavailability, which depends on a number of dietary, as well as host-related factors that lead to inter-individual variability in absorption and metabolism. These factors include diseases (e.g., malabsorption, inflammatory bowel disease, etc.), lifestyle habits (e.g., smoking, drinking, etc.), gender and age, as well as carotenoid metabolism (Table 1) [16]. Of note, one key dietary factor affecting L and Z bioavailability is the presence of fats in a meal. As these are lipophilic molecules, the type (i.e., monosaturated versus polyunsaturated) and amount of fat in the diet, are paramount to ensure the solubilization and absorption of carotenoids, with polyunsaturated fat more likely to enhance L or Z intestinal uptake.[26] In contrast, presence of dietary fiber affects the release of XCs from the food matrix. In addition, XC are poorly released from raw vegetables due to the solid structure of the cell walls, interfering with the release of carotenoids into the gastrointestinal lumen.

**Table 1:** Factors affecting L and Z Bioavailability

Host-related factors	Food-intrinsic factors
Diseases: malabsorptive disorders (e.g., pancreatic insufficiency, celiac disease) and inflammatory conditions (e.g., inflammatory bowel disease)	Food composition: different food matrices (e.g., dense matrices in green leafy vegetables)
Medicines: ezetimibe	XC content
Lifestyle habits: smoking and alcohol intake	Seasonal produce
Sex and older age	Geographic location and climate
Single nucleotide polymorphisms (SNPs) and genetics	Stage of maturity when harvested and growing conditions
Dietary patterns (e.g., combination of lipids and dietary fibers)	Cooking, processing, and preservation methods
Race and ethnicity: main food sources	Devolution

From a clinical perspective, preventive and therapeutic effects of L, Z, and MZ remain a subject of great interest. In the area of human nutrition, establishing causal relationships between nutrient intake and its effects in human health is a complex. Additionally, the ongoing journey to understand the mechanisms governing the absorption, transport, bioconversion, and excretion of L, Z and MZ is challenging, [17]. Future studies are needed to support individuals at risk of reduced delivery of L, Z and MZ that could benefit from targeted nutritional recommendations. Nonetheless, the known intrinsic and extrinsic dietary factors of the XCs allow for the development of targeted nutrition guidelines. The increasing worldwide incidence of cardiometabolic diseases (CMDs) with abnormal lipid metabolism poses additional challenges for adequate intake of the XC. The therapeutic management of dyslipidemia, which includes ezetimibe, a first line hypolipidemic drug that inhibits SRB1 and has been shown to decrease Z absorption [39].

By consistently incorporating these carotenoids into one’s diet or considering targeted supplementation, individuals can maintain optimal levels over time, resulting in sustained protective effects and potentially preventing or delaying the onset of vision-related diseases, as well as chronic and degenerative conditions like CMDs. Emphasizing preventive nutrition approaches encourages individuals to adopt healthier lifestyle habits. Promoting the consumption of xanthophyll carotenoid-rich foods, such as leafy greens, citrus fruits, and colored vegetables, encourages a well-rounded, nutrient-dense diet. This approach supports overall health and may have additional benefits beyond eye health, such as cardiovascular protection and cognitive function. Taking a clinical perspective to address factors like the limited bioavailability of XC can enhance their integration into healthcare and public health directives. One plausible strategy to tackle these considerations is by classifying factors that affect L and Z bioavailability as either modifiable or non-modifiable, which will likely pinpoint areas of opportunity for targeted nutrition. This novel classification not only facilitates scientific discourse but also bridges the communication gap between researchers, healthcare practitioners, and patients.

Incorporating nutritional or antioxidant biomarkers into clinical practice has the potential to offer a comprehensive perspective on the underlying mechanisms of CMDs, which currently stand as the most prevalent conditions and a leading contributor to mortality worldwide [95]. By adopting this approach, healthcare practitioners could integrate these biomarkers into the diagnostic framework of CMD cases. These outcomes will inform the revision of local guidelines for the prevention and management of specific chronic conditions. This endeavor, which requires a multidisciplinary approach that implements lifestyle changes and a holistic therapeutic management [125].

## Conclusion

The annual CRN-International Scientific Symposium reflected upon the health challenges resulting from malnutrition and an aging population, both that come with significant social and economic costs. The five recognized experts shared their perspective on the importance of focusing on prevention and optimizing nutrient status prior to the onset of health-related issues. Recognizing that the globally agreed nutrition goals are off-track and healthy diets are not affordable or accessible to all, there is an urgency for the evolution in policy and research to enable forward progress.

Nutrition policy recommendations to prevent nutrient deficiencies remain important, however, efforts should evolve to consider recommendations that support resilience, optimal health and expanded healthspan. Going beyond nutrition, policy shifts are needed across multiple sectors to enable households and individuals to consume a healthy diet and ensure those most vulnerable to malnutrition are provided with access to them. Suggested solutions included; (1) transformation of agriculture and trade policy to prioritize actions to ensure availability and access to nutritious food, (2) protection of policy continuity gains from political interests taking precedence over prioritized programs that ensure nutrition actions in the context of universal health care and effective social protection, and (3) incentives and disincentives to shift food production towards healthier food to address the many nutrition issues linked to the high availability and lower cost of unhealthy foods.

Solutions to address these health challenges and policies must be based on reliable evidence. Evaluation of the impact and cost effectiveness of these actions are necessary, requiring innovation in methodological approaches, moving away from reliance on the medical models (i.e., randomized trials of single nutrient interventions), that are not feasible or appropriate. Advancing research to identify better biomarkers for optimal health and healthspan along with the factors that influence individual response variability are essential to inform future public and personalized recommendations.

The opportunities identified start to build a roadmap for impact, but action is required. The decisions that are made today to progress nutrition science and policy will design the future for the next generation. Therefore, it is critical that all stakeholders (government, academia, private sector) come together to identify and implement solutions that will optimize nutrition status and improve healthspan to enable heathier lives, perhaps going beyond traditional public health measures.
